# Rationale for integration of services for diabetes mellitus and diabetic retinopathy in Kenya

**DOI:** 10.1038/s41433-022-02000-x

**Published:** 2022-05-19

**Authors:** Nyawira Mwangi, Covadonga Bascaran, Stephen Gichuhi, Mathew Kipturgo, Lucy Manyara, David Macleod, Consuela Moorman, Allen Foster

**Affiliations:** 1 Kenya Medical Training College, Nairobi, Kenya; 2 London School of Hygiene & Tropical Medicine, London, United Kingdom; 3 Department of Ophthalmology, University of Nairobi, Nairobi, Kenya; 4 Oxford University Hospitals NHS Trust, Oxford, UK

## Abstract

**Background:**

Good diabetes mellitus (diabetes) and diabetic retinopathy (DR) management depends on the strength of the health system, prompting us to conduct a health system assessment for diabetes and DR in Kenya. We used diabetes and DR as tracer conditions to assess the strengths and weaknesses in the health system, and potential interventions to strengthen the health system. In this paper, we report on the need and relevance of integration to strengthen diabetes and DR care. This theme emerged from the health system assessment.

**Methods:**

Using a mixed methods study design, we collected data from service providers in diabetes clinics and eye clinics in three counties, from key informants at national and county level, and from documents review.

**Results:**

There is interest in integration of diabetes and DR services to address discontinuity of care. We report the findings describing the context of integration, why integration is a goal and how these services can be integrated. We use the results to develop a conceptual framework for implementation.

**Conclusions:**

The principal rationale for integrated service provision is to address service gaps and to prevent complications of diabetes and DR. The stakeholder interest and the existing infrastructure can be leveraged to improve these health outcomes.

## Introduction

Integration of services is a strategy for improving the performance of health systems and achieving clinical outcomes. The World Health Organization’s (WHO) working definition of “integrated service delivery” is: “the management and delivery of health services so that clients receive a continuum of preventive and curative services, according to their needs over time and across different levels of the health system.” [1] This definition posits integration as a composite construct with continuity and quality of care being essential components. The aim is to provide services that meet the needs of the user: services that are not disjointed, that are easy to navigate and that provide a smooth link to specialist services, if required [1, 2]. Although there is consensus on the desirable outcomes of integration, and its importance for universal health coverage in every country, the rationale and the operational models remain contextual [3, 4].

Diabetes mellitus (hereafter referred to as ‘diabetes’) is associated with the development of organ damage, leading to multiple morbidity. Providing care for people living with diabetes (PLWD) thus requires balancing diabetes management with management of its chronic complications. Diabetic retinopathy (DR) is the major ocular morbidity in diabetes, and there is strong epidemiologic evidence that its prevalence is increasing [5, 6]. In common with other chronic diseases, the management of both conditions requires: promotion of healthy lifestyle, early detection, compliance to treatment, regular monitoring of treatment outcomes, active involvement of the patient and family in the care. An integrated approach is an efficient and effective method of addressing inter-related chronic diseases [4, 7, 8]. At present, diabetes and DR care are provided in diabetes and eye clinics respectively, with minimal collaboration between them. Given that the patient with DR also requires diabetes services, and the similarities in the approach to the management of both conditions, it is appropriate to explore the extent to which diabetes and DR services are provided in a comprehensive or integrated manner [9]. This provides an opportunity to consider how integration would intersect with the need for specialist services.

The literature on integration of diabetes services has largely focused on integration with HIV, tuberculosis and hypertension [8, 10–12]. The paucity of literature on integration with services for DR services might be based to the assumption that these services automatically integrated, since they are intricately linked. However, the point of entry into integration is often unspecified. Further, the interventions that should be integrated, and in which ways and by whom, is not explicit even in clinical guidelines. The evidence on what diabetes practitioners and eye care practitioners think of the integration, or of their professional relationship is also sparse [13].

Proactive prevention and early detection of DR is an important best practice that is often missing in the services for PLWD. As this population has regular contact with diabetes services, this platform is a good entry point to bring DR services to where the patient is, or to link the patient to the eye service, where the DR services are provided. Innovative approaches such as integration can augment access, quality and continuity of care for PLWD. In this paper, we explore the interface between diabetes services and eye care services in Kenya, as an unexploited area for integrated care for DR. We use our results to develop an operational framework for integration.

## Methods

### Study design and theoretical approach

A mixed-methods cross-sectional health system assessment for diabetes and DR was conducted in three counties of Kenya, guided by the WHO’s health system building blocks framework and the tracer condition approach [14, 15]. The aim of focusing the assessment on diabetes and DR was to provide evidence relevant to services for the two conditions, which may be missed in a general health system assessment. Both conditions meet the criteria for a tracer condition (Table 1). In line with the WHO framework, we defined a good service as one which delivers effective, safe, quality, personal and non-personal health interventions to PLWD, when and where needed, with minimum waste of resources [16]. In this paper, we report on integration as a theme that emerged from the assessment.

**Table 1:** Diabetes and DR as tracer condition.

Criteria	How diabetes and DR fit the criteria
Disease has a known epidemiology	The epidemiology of both conditions has been described
Disease is well defined and easy to diagnose	The definition and criteria for diagnosis is well-established
Its prevalence in the population is large enough to enable adequate data to be collected	Both population-based and clinic-based surveys have shown that that the prevalence is sufficient to enable collection of data that can be used for planning services
Its natural history is known, and it varies with the utilisation and effectiveness of health care	The natural history including the predictors of the development of complications is known
It requires specific treatment, in the absence of which functional impairment results	Hypoglycaemic drugs and lifestyle measures are required for glycaemic control, and the treatments for DR have been described, without which visual impairment results
Available and well-defined techniques of medical management exist for at least one of the following: prevention, diagnosis, treatment or rehabilitation	Prevention, diagnosis and treatment apply to both conditions. Rehabilitation is provided for those who develop severe visual impairment and blindness

### Sampling and data collection

Kirinyaga (predominantly rural), Nakuru (semi-urban) and Nairobi (urban) counties were selected through stratified purposive sampling to represent these different regions within the diabetes belt in Kenya. Three health facilities providing outpatient diabetes services in each county were identified by simple random sampling from a sampling frame of the clinics. Two clinicians who provide diabetes services were interviewed (*n* = 3 counties*2 clinicians*3 facilities = 18). Three eye care workers providing services in the county were also interviewed (*n* = 3 workers*3 facilities = 9). The primary investigator and research assistants interviewed the 27 service providers at the clinics using a structured questionnaire with both closed-ended and open-ended questions.

Key informants (*n* = 18) at national and county level were interviewed. We defined key informants as representatives of stakeholders in the diabetes and eye care services, who were familiar with the organisation and delivery of healthcare at the national or at county level, but whose principal role in the health system is non-clinical. Key informants were initially identified using a sampling frame and subsequently through snowballing from those interviewed, until data saturation was reached. Those interviewed included eight health service managers, four non-governmental organisation (NGO) programme leaders, four policy makers and two members of the umbrella PLWD body that represents patients. The primary investigator interviewed the key informants at their work sites or preferred locations using a topic guide. Interviews lasted 45–60 min, were audio-recorded and extensive field notes were taken. The data collection instruments had questions on the strengths and weaknesses of the health system for diabetes and DR, and potential interventions to strengthen the health system. We also conducted document review of health system documents provided by the key informants and service providers.

### Ethics

The London School of Hygiene & Tropical Medicine and African Medical Research Foundation (AMREF) granted ethical approval. All participants gave written informed consent. Participation was voluntary and participants did not receive any financial incentives.

### Data analysis

Audio records were transcribed verbatim. All textual data (from interviews and documents) were analysed using thematic content analysis, and guided by the theoretical frameworks [17]. The primary investigator and a second independent coder read and summarised the interviews to get an overview of potential themes. Where clarifications with participants were required, they were contacted on telephone. The coders discussed and agreed upon a coding structure before coding the transcripts section by section independently. The codes were grouped into subthemes and subsequently collapsed into themes within the six building blocks of the health system. We reviewed themes repeatedly across all transcripts. Quantitative data and data from document review were summarised using descriptive statistics and summary tables respectively. Triangulation of different types and sources data was useful for elaboration and providing complementary insights.

## Results

### Characteristics of participants

We interviewed 18 key informants and 27 service providers from diabetes services (*n* = 18) and eye care (*n* = 9). None of the participants invited declined to participate. Of the 45 participants, 25 (56%) were male, the median age and duration of employment being 41 years and 15 years respectively. We examined 22 documents, which were strategies and strategic plans, reports, policies, published literature, and meeting presentations related to diabetes and DR in Kenya.

### Integration as an emerging theme in participant interviews

When the participants were prompted to discuss potential interventions to strengthen the health system for diabetes and DR, integration emerged as a dominant theme. Table 2 shows sample quotes within this theme.

**Table 2:** Integration as a theme in the different building blocks.

**Leadership and Governance**
*We have been working very closely (with ophthalmic services) at national level…the next step is integration of diabetes eye care services into comprehensive diabetes services (Key informant, diabetes care)*
*Our policies, which include DR care, fit into the NCD policies…but in practice they do not seem to work in an integrated way (Key informant, eye care)*
**Service delivery**
*We offer a wide range of services in the diabetes outpatient clinics…but there is a missing link with the eye services…you know, for the annual eye examination (Key informant, diabetes care)*
*Eye care services for DR are part of the wider community of diabetes services, and also part of the wider community of eye care services (Key informant, eye care)*
**Human resources for health**
*We need integration of the training on comprehensive diabetes management…we have to integrate the eye component into it, and we have to integrate this training in the preservice curriculum of colleges and universities…this is actually a low-hanging fruit (Key informant, diabetes care)*
*Nurses in the diabetes clinic and in the eye clinic should also be trained as trainers of trainers in diabetes eye care…they need to be part of the team (Service provider, eye care)*
**Medicines and health technologies**
*NHIF [National Hospital Insurance Fund] caters for the costs of inpatient care for both diabetes and DR, now we need to include all tests and medicines for both conditions in this cover (Key informant, eye care)*
*We value integration of services,…it may help to ensure we don’t lose PLWD to informal services… we would not integrate herbal medicine into our services, but this is a cultural and social issue that cannot be addressed by us alone (Key informant, diabetes services)*
**Health Management Information System**
*Surveillance of chronic illnesses like diabetes and DR is difficult. The solution is an integrated electronic medical records system (Key informant, diabetes care)*
*Even though we do not use the same reporting system or software, it should still be possible for us to have access to relevant data from the diabetes system, and vice versa…. we are not talking of a merger (Key informant, eye care)*
**Health financing**
*I would suggest that an integrated implementation framework be developed at the county level, and it should have a dedicated budget (Service provider, diabetes care)*
*DR being principally a diabetes issue, we need to present the case for financing for DR by NHIF as part of diabetes services (Key informant, eye care)*

### Integration as envisaged in government policies and plans

Integration is a key policy objective as reflected in a sample of the documents, Table 3. Possible integration with HIV, Tuberculosis and Malaria programmes is envisaged, though how it should be done is not explicit. Integration of diabetes and DR is not mentioned.

**Table 3:** Examples of concepts of integration in a sample of health system plans and strategies.

**Kenya Health Sector Strategic and Investment Plan 2018-2023**
*The Strategic Plan uses an integrated people-centered approach to service deliver services (page 49)*
**Kenya Essential Package for Health**
*Institutional screening for NCDs is one of the KEPH interventions for reversing the rising burden of NCDs. The services targeted are routine BP, routine BMI and blood sugar testing*.
**Kenya National Strategy for the Prevention and Control of Non-Communicable Diseases 2015-2020**
*Several bottlenecks of non-communicable disease (NCD) prevention and control have been identified and addressed in this strategy, including: “Silo” nature of the health system with minimal opportunities of integrating NCDs in well-established public health care platforms like HIV, TB, family planning, maternal and child health. (Page 31 and 32)*
*Strategic Objective 1 of the strategy: To establish mechanisms to raise the priority accorded to NCDs at national and county level…The interventions for this objective include integrating NCD prevention and control into policies across all government sectors*.
**Kenya National Diabetes Strategy**
*The objectives of the Kenya diabetes strategy include:*
*• To improve early detection for diabetes and its complications through screening*
*• To network and integrate diabetes care with other national programmes e.g. HIV/AIDS, TB and malaria (Page 9)*
*One of the activities in the resource mobilisation strategy is:*
*• Integrate diabetes prevention and control into the health plans (Page 11)*
**Kenya Service Availability and Readiness Assessment Mapping (SARAM) Report**
*• General service readiness for provision of NCD services is 34% (for the KEPH-defined NCD services)*
*• There is an overall limitation on the availability of KEPH services contributing to reversing the burden of NCDs (Page 112-126)*
**Norms and Standards for Health Service Delivery**
*Integration of care: Every contact with individuals, households and communities is used to ensure that a comprehensive set of defined services is made available. This is different from using “every opportunity to do everything”. (Page 4)*

### How integration of diabetes and eye care services can be implemented

Participants described a positive existing relationship between diabetes and eye care services in the context of DR, and envisioned a closer and newer way of ‘mutual accommodation’:

In the DR-NET [Diabetic Retinopathy network] programme, we have worked very well as physicians and ophthalmologists (Key informant, diabetes service)

Diabetes services need to accommodate us more, it seems that DR gets forgotten (Service provider, DR)

We identified three points of emphasis regarding how the integration of the two services should be implemented. Firstly, is that DR should be integrated into diabetes services. This is because of the pre-requisite for a functional service, such as the diabetes services, to which the DR service can be integrated. Policy documents recognise that services should be integrated into existing well-established health services or programmes, Table 3.

Sometimes we forget the eye, because there are too many different things that have to be done for the patient…” (Service provider, diabetes)

We would like DR to be seen as a diabetes issue, not an eye care issue (Key informant, eye care)

When we review the diabetes guidelines, DR will take centre stage (Key informant, diabetes service)

Of the 18 key informants, 17 (94.4%) believed that DR services should be integrated with diabetes care. 61.1% of key informants (*n* = 11) reported that diabetes services should lead in the integrated service because they have a stronger infrastructure and accessibility to PLWD. However, 33% of key informants (*n* = 6) indicated that eye care infrastructure in some hospitals is stronger than the diabetes infrastructure, but diabetes services should lead the integration because they have a stronger reach to the PLWD. One key informant (5.5%) felt that the discourse on the relative merit of integration should not focus on the infrastructure but should strengthen links between the services.

The second point of emphasis is that eye care workers have a role in enhancing care for diabetes, as well as care for other non-communicable diseases:

Using the eye examination, eye clinicians can monitor diabetes and hypertension…because the finding of diabetic or hypertensive retinopathy is useful information (Key informant, diabetes service)

Eye care workers should ask patients about diabetes control (Service provider, diabetes)

Thirdly, both diabetes and eye care services need to work together:

Sometimes they {eye care services} will just examine the eye and not be interested in the medical management of the diabetes…we should all be seen to be involved with this (Service provider, diabetes)

Those of us on the ground…we know that DR is being missed in diabetes services…I think we need to go to the diabetes clinic…get involved with diabetes and get to look into the eye (Service provider, DR)

Key informants identified that referral and screening for DR might be strengthened through integration:

Although we have links with diabetes services, there are unexploited opportunities to integrate DR, particularly screening, with routine diabetes services (Key informant, eye care)

There is no follow up system so that even if a diabetes patient is referred to eye clinic and disappears, there is no system of follow up or feedback, as currently we work as separate services (Service provider, diabetes)

Of the 18 diabetes clinicians, only one had received a patient referred from eye services in the preceding month, while four of the nine eye care clinicians had received a referral from diabetes services in the same period. This implies that cross-referral is ineffective; there are missed opportunities for referral or patients are lost in transit. Furthermore, none of the diabetes clinics had visual acuity charts or ophthalmoscopes, and none of the eye clinics had a functional glucometer, which shows there is a big role for referral. The lack of readiness of health facilities for provision of NCD services is also noted in the Service Availability and Readiness Mapping (SARAM) report, Table 3.

### Benefits of integration

We identified three main benefits of integration. First, integration can help to address service fragmentation, Table 3, as well ensure that patients access all the services they need. Participants suggested that integration may provide opportunities for joint on-the-job training for staff, which is a priority because 12/18 diabetes clinicians and 4/9 eye care clinicians had not had a recent training update on DR and diabetes respectively. Secondly, participants suggested that integration might enhance continuity of care and increase awareness of DR among diabetes care providers. Thirdly, participants also identified that integration can attenuate potential problems, such as conflicting clinical recommendations that confuse PLWD and staff. However, none of the participants suggested that integration would have an economic benefit.

### Steps towards implementing integration

We found that the policy documents do not elaborate how integration should occur. However, the norms and standards document states that integration “*does not mean’doing everything’…”*. This implies the need to establish the priorities. The participants identified four main priorities: referral (*n* = 34), retinal screening for DR (*n* = 23), patient monitoring (*n* = 19) and patient education for self-management (*n* = 16). Seven participants remarked that the interaction between diabetes and eye clinics must be continuous, particularly through bidirectional referral; otherwise, “*integration will be ineffective*”. Five participants indicated that the integration should be gradual, and preceded by a pilot.

The inputs that will be needed to achieve integration were listed: joint planning, joint training of health workers on diabetes and DR, equipment for monitoring diabetes (glucometer, test strips), DR screening equipment, a database that includes both diabetes and DR, clinical checklists and guidelines. Key informants suggested that financing for the additional inputs and processes would be sourced from the government and partners. All diabetes clinicians indicated that they would be happy to have a retinal camera situated in their clinic, though they had space constraints. Six of the nine eye care service providers were willing to hold regular outreach clinics to screen PLWD for DR.

**Fig. 1:**
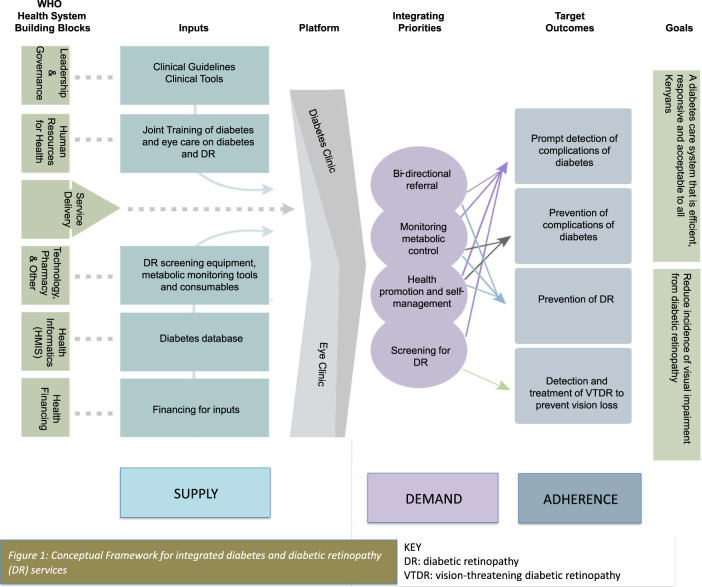
Conceptual framework for Integrated diabetes and diabetic retinopathy services. Inputs required to make integration feasible and outcomes envisaged in successful integration.

All participants concurred that they would have roles in the integration, which include; getting buy-in from all staff and administrators, facilitating or participating in joint planning, obtaining the resources and supporting implementation. Participants identified that integration should be led by the team leads in diabetes and eye clinics, and should prioritise strengthening referral, metabolic control, self-management, and screening for DR. Based on the findings in this study, we present a conceptual framework for integration (Fig. 1).

## Discussion

Integrated health systems have been promoted as a means to build a more effective, efficient and patient-focused health system that better meets the needs of the populations served [4]. Integrated DR services can blur the boundary between diabetes services and eye care services, to create a shared repertoire and synergy for the investments made in these services. Such synergy is vital for strengthening the health system responsiveness to the rising burden of diabetes and DR [7, 13]. It can ensure equity by reducing the exclusion and difficulty in navigating the services by PLWD, since comprehensive diabetes care would include DR services. Further, it provides a unique opportunity to integrate primary, secondary (early detection) and tertiary prevention (treatment to prevent complications), Fig. 1. This can lead to improved health outcomes and therefore more cost-effective use of health system resources by PLWD. For example, screening and detection of DR might improve adherence to self-management and optimal metabolic control, which would prevent additional complications of diabetes that require expensive treatment.

The endorsement of integration in the health policies is relevant to its sustainability, because it implies long-term government commitment [7]. Integration of comprehensive diabetes care with HIV, tuberculosis and malaria services would entail investment at all levels of health care, as services for these communicable diseases are offered across the continuum of primary, secondary and tertiary care. To ensure that comprehensive diabetes care includes DR, we propose a service-level model of integration at the diabetes clinics and eye clinics, which are usually located at secondary and tertiary hospitals. This is expedient for three reasons. One, the resources for integration are already available at this level of the health system, where the two clinics are already functioning. This would avoid aggravating existing resource challenges, such as health workforce shortages [18]. Two, the integrated service removes the complexity that patients face while navigating the care pathway, which often presents a barrier to access to DR services [1, 19]. Three, the integrated services includes specialist diabetes and eye care services, which shows that integration does not imply compromising specialist functions [1, 2]. Such a fear can cause resistance by specialists, although this was not evident in our study [1, 19].

Furthermore, there was high level of interest on integration among all participants, which is likely to facilitate successful implementation of integration. This is important because reluctance, opposition or lack of ownership by the service providers would lead to poor integration results [20]. In other studies, service providers have been concerned about the likely increase in workload [11, 20]. In this study, staff shortage, inadequacy of space for additional services, lack of equipment and weak referral linkage were identified as potential challenges but not as deterrent to the integration. Although we did not investigate the reasons for this enthusiasm, it might be because diabetes and eye care services target the same population (PLWD) and have a converging goal in relation to DR (prevention of blindness). It might also have resulted from several system antecedents: (1) An ongoing pilot programme of the DR-NET hospital-twinning initiative, which is a LINK programme involving both diabetes and eye care stakeholders, with the aim of strengthening DR services. (2) A recent national STEPwise survey for risk factors of non-communicable diseases, and (3) Sensitisation of participants on DR as a potentially blinding condition.

The reasons for integration nominated by participants reflect the perceived differences between integrated and non-integrated care. The main impetus is local service gaps, such as fragmentation, and missed opportunities for early detection of DR or inefficiency and discontinuity of care, which concurs with the drivers cited in other literature [2, 7, 10, 13, 20]. These are typical barriers to access to care that will be addressed through integration [2]. The necessity for integration has also been recognised in a previous study in Kenya [13]. Cost-control was not identified as the major driving force for integration in this study, unlike in other contexts [19]. However, integration is likely to reduce costs by reducing duplication of services and multiple client visits [1, 12, 20]. In addition, early detection or DR is a sound economic investment because timely treatment is cost-effective [21, 22]. This shows that the interest in integration among these participants was predicated on improved services outcomes and not as an end in itself.

The conceptualisation of integration around screening is significant because Kenya does not have a systematic screening programme for diabetes or DR [13]. It reveals an excellent opportunity to develop an effective screening programme inclusive for all PLWD attending diabetes services. The bidirectional referral strategy shows the pertinence of organising integration as a process of mutual but not symmetric accommodation. It is not symmetric because the entry point is the diabetes services, which PLWD are already accessing even without integration; hence, it is the primary service. An excellent example of how synergy might be realised is that eye care workers can identify and monitor comorbidities. Ocular findings in hypertension, hyperlipidaemia, and other medical conditions may be the first sign of these diseases, and these can be identified during the screening examination. Medications used to treat these comorbidities might also have ocular adverse effects that can be identified upon ocular examination. A comprehensive dilated eye examination can be a radar for detection or monitoring several comorbidities and medications.

The integration could be operationalised through co-implementation of the key interventions, which are self-management, glycaemic control, DR screening and referral. This scope focuses on prevention of complications, rather than treatment [23]. Some integration models, such as sexual and reproductive health programmes integrating into HIV programmes, have focused on clinical services, such as testing or prescription of treatment, rather than lifestyle modification [11, 24]. Conversely, other integration models for diabetes, HIV and hypertension have emphasised on adherence counselling for medication and lifestyle modification [25]. Still, other models have included a mix of patient education and prescription of medications for PLWD [10, 26, 27]. No method is inherently good or bad, the scope largely depends on the objectives of the two services [1, 7, 8]. Given the priority for diabetes and DR is to prevent progression or complications, bidirectional referral and health promotion approaches would be useful [13, 21]. In the event of future integration with HIV, Tuberculosis and Malaria programmes, a treatment component may be added, since the main priority for these conditions is universal coverage to treatment for those eligible [7, 27].

Several inputs are required: inter-professional collaboration, joint planning, clinical governance, training, clinical tools, database and equipment. This means that supply-side resources are required, and need to be allocated differently [7, 13]. Integration is not monolithic but encompasses all building blocks of the health system and hence requires resource mobilisation. Given that integration cannot mitigate against lack of necessary resources or infrastructure, failure to invest in it would hamper the desired benefits[1, 23]. For example, the lack of a monitoring and evaluation component has been identified as a weakness in previous integration initiatives [28]. This being one of the first studies to discuss this context of integration, we have proposed a conceptual framework for integration, which can be used by policy-makers for planning Fig. 1.

What are the expected effects of integration? Investing in this integrated service delivery system creates distinct deliverables, such as increased demand for the specified services and reducing the unmet need for DR screening [7, 12, 21]. Integration should translate to prevention of complications of diabetes and DR, which is a widely agreed priority of health systems [21]. To monitor whether integration confers these benefits, an appropriate metric will need to be jointly determined.

Our study has several strengths. Geographical variability (three counties) accentuated the external validity of the study. The inclusion of clinicians from both diabetes and eye care services, as well as patient representatives, enabled us to obtain unique perspectives of service providers. The data is subject to social desirability bias as the participants are directly involved in the services, however we used triangulation to mitigate this. This is the first study to document the interventions and the platforms for integration of these services in the region. The main limitation is the novelty of the concept of integration with respect to diabetes and DR but this shows that this health system is dynamic, and it may jumpstart the process of broader integration of diabetes services.

## Conclusion

Integration, as envisaged in this paper, is relevant to the goals of the health system and congruent to the existing health system for diabetes and DR and to the broader health strategies in Kenya. The purpose of integration is to address service gaps, ensure universal access to a range of services and prevent complications of diabetes and DR. This is evidence that the health system is not static in its response to NCDs, and integration may be applicable to other countries with similar health systems.

### Future research

Further research is required to test and refine this empirical conceptual framework. The impact of integration on the following parameters will also need to be evaluated: performance of health workers, service utilisation, patient satisfaction and cost of the services. The effect of integration on equity may be determined by disaggregating the health outcomes of PLWD by gender, social strata and other indices of vulnerability.

## Summary

### What is known about this topic

- There is growing recognition and advocacy for integrated care as an intervention for strengthening health systems for non-communicable diseases.
- Despite a growing body of research and models on integration, there are significant knowledge gaps in the literature on the feasibility of integration of care, particularly in LMICs.
- Context-specific evidence to adapt integrated care for diabetes and diabetic retinopathy to fit the needs of specific counties is required.

### What this study adds

- This is the first study to provide evidence on the rationale for integration of diabetes and diabetic retinopathy services in Kenya, using a health system lens.
- The degree to which the need for integration is reflected in health policy documents in Kenya is explored.
- A conceptual framework for integration of services in Kenya is provided.
- Potential facilitators and barriers to the implementation of integrated services have been elicited.

## Data Availability

The data that support the findings of this study are available from Ministry of Health, Kenya but restrictions apply to the availability of these data, which were used under license for the current study, and so are not publicly available. Data are however available from the authors upon reasonable request and with permission of the Ministry of Health.
